# Keratocan Improves Muscle Wasting in Sarcopenia by Promoting Skeletal Muscle Development and Fast‐Twitch Fibre Synthesis

**DOI:** 10.1002/jcsm.13724

**Published:** 2025-02-17

**Authors:** Xu Chen, Yanyan Zhang, Zhibo Deng, Chao Song, Linhai Yang, Rongsheng Zhang, Peng Zhang, Yu Xiu, Yibin Su, Jun Luo, Jie Xu, Hanhao Dai

**Affiliations:** ^1^ Shengli Clinical Medical College of Fujian Medical University Fuzhou China; ^2^ Department of Minimally Invasive Spinal Surgery The Affiliated Hospital of Putian University Putian China; ^3^ Department of Clinical Laboratory The Affiliated Hospital of Putian University Putian China; ^4^ Department of Orthopedics Fujian Provincial Hospital Fujian Medical University Fuzhou China; ^5^ College of Traditional Chinese Medicine Fujian University of Traditional Chinese Medicine Fuzhou China

**Keywords:** keratocan, osteoporosis, osteosarcopenia, PI3K/AKT/mTOR pathway, sarcopenia

## Abstract

**Background:**

Osteosarcopenia refers to the co‐occurrence of osteoporosis and sarcopenia, which are characterized by progressive bone density and muscle mass loss, respectively. Muscle and bone are regulated by many common genes and pathways, enabling potential co‐treatment. Because keratocan protects against osteoporosis, we hypothesized it may also protect against sarcopenia, implying a new co‐intervention target. This study aimed to elucidate the role and molecular mechanisms of keratocan in skeletal muscle.

**Methods:**

We analysed keratocan expression in the muscles of aged mice and patients with osteosarcopenia and during the differentiation of C2C12 myoblasts. The regulatory role of keratocan was assessed by knocking down or overexpressing keratocan in C2C12 cells and examining any effects on myogenic proliferation and differentiation. RNA sequencing analysis was also performed on these cells. The relationship between keratocan and enriched signalling pathways was verified using pathway inhibitors or agonists. Finally, adeno‐associated virus‐9 containing a muscle‐specific promoter was injected into SAMP8 senile mice to observe the effects of keratocan overexpression.

**Results:**

Keratocan expression was significantly lower in the skeletal muscles of aging mice (−2.02‐fold, *p* < 0.01) and patients with osteosarcopenia (−1.78‐fold, *p* < 0.001) compared with that in controls. Keratocan overexpression resulted in a significant increase in the proliferation indices CCND1 (+1.43‐fold, *p* < 0.001), Ki67 (+2.30‐fold, *p* < 0.001) and PCNA (+1.975‐fold, *p* < 0.01) and the differentiation indices MyoD1 (+2.156‐fold, *p* < 0.001), MyoG (+1.52‐fold, *p* < 0.05) and myosin heavy chain (MyHC; +2.849‐fold, *p* < 0.01); conversely, the muscle atrophy indices MuRF‐1 (−30%, *p* < 0.01), atrogin‐1 (−87%, *p* < 0.01) and myostatin (−24%, *p* < 0.01) were significantly decreased. PI3K/AKT/mTOR was identified as a potential pathway for keratocan regulation in C2C12 cells. PI3K inhibitor LY294002 reversed the promotion of myogenesis by keratocan overexpression, while PI3K activator 740Y‐P reversed the inhibitory effect of keratocan knockdown on myogenesis, promoting myofibre development and ameliorating muscle atrophy in SAMP8 aging mice. This was evidenced by increased mean muscle cross‐sectional area (+38%, *p* < 0.0001) and muscle mass (+7%, *p* < 0.01) and decreased fibrosis (−40%, *p* < 0.01). Furthermore, keratocan facilitated the conversion of slow‐to‐fast muscle fibres through the PI3K/AKT/mTOR pathway, characterized by significantly increased grip strength (+42%, *p* < 0.01) and maximum running speed (+19%, *p* < 0.001), and decreased fatigue time (+13%, *p* < 0.05).

**Conclusions:**

Keratocan ameliorates muscle atrophy by activating the PI3K/AKT/mTOR pathway, promoting muscle satellite cell proliferation and myogenic differentiation, and facilitating the conversion of slow‐to‐fast muscle fibres. Our findings demonstrate the potential of keratocan as a novel therapeutic target for osteosarcopenia.

## 1 Introduction

Sarcopenia (SP) is an age‐related progressive skeletal muscle disease characterized by the loss of skeletal muscle mass and function [1, 2]. Osteoporosis (OP) is a systemic bone disease characterized by decreased bone density and mass and the destruction of the bone microstructure, resulting in increased bone fragility and fracture susceptibility [3]. As life expectancy increases, the prevalence of osteoporosis and sarcopenia increases, often occurring simultaneously, termed osteosarcopenia (OS) [4, 5]. Compared with sarcopenia or osteoporosis alone, OS has a higher rate of disability and mortality in elderly patients, imposing a heavy burden on patients and society [6]. Muscle and bone are not only adjacent in location and complementary in function, but both originate from mesenchymal stem cells [7], with homology in tissue development. Muscle and bone are regulated by many common genes and signalling pathways, suggesting the possibility of sarcopenia and osteoporosis co‐treatment [8, 9]. However, the common therapeutic targets and mechanisms of sarcopenia and osteoporosis are unclear, and there are currently no drugs available to treat osteosarcopenia.

With advances in sequencing technology, bioinformatic analysis has played an increasingly important role in target discovery [10]. Our bioinformatics analyses showed that keratocan expression was reduced in the muscles of sarcopenia mice and the bones of osteoporosis mice, suggesting that keratocan may be a common protective gene. Keratocan belongs to the small leucine‐rich proteoglycan family, members of which are known to play a role in regulating cellular processes [11, 12, 13]. Igwe et al. [14] showed that keratocan is highly expressed in mature osteoblasts and promotes osteoblast differentiation, which is consistent with our bioconvection results; however, the role of keratocan in sarcopenia remains unclear. In this study, we examined keratocan expression in the skeletal muscle of aged osteosarcopenia mice and patients and overexpressed or knocked down keratocan in C2C12 cells to investigate the effects and molecular mechanisms on myosatellite cell proliferation and myogenic differentiation. Further, gastrocnemius muscle injections using the muscle‐forming specific promoter adeno‐associated virus AAV9‐oe‐keratocan in a rapidly aging SAMP8 mouse animal model were used to explore the clinical value of keratocan in delaying and treating sarcopenia.

## 2 Methods

### 2.1 Bioinformatics Analysis

Gene expression data were obtained from the Gene Expression Omnibus (GEO) database (GSE209528, GSE175562, GSE213148, GSE186104 and GSE202395; http://www.ncbi.nlm.nih.gov/geo/). The sample characteristics are listed in Table S1. Differentially expressed genes were identified using the Limma R package. In this study, genes with a *p*‐value < 0.05 and a multiplicity of change > 1 were considered DEGs. The Venn diagram tool website (http://bioinformatics.psb.ugent.be/webtools/Venn/) was used to identify overlapping genes.

### 2.2 Immunohistochemistry (IHC)

Muscle samples were stored in chilled saline, fixed in 4% paraformaldehyde for 24 h, and transferred to a 70% ethanol solution. The samples were then dehydrated through a graded ethanol series; the ethanol was removed with xylene, and the tissue samples were embedded in paraffin. Paraffin‐embedded muscle tissue sections were deparaffinized, dehydrated and incubated with primary antibody at 4°C overnight. They were then incubated with secondary antibodies, followed by incubation with DAB horseradish peroxidase chromogenic kit (P0202, Beyotime, China) according to the manufacturer's instructions, and stained with haematoxylin for nuclei observation. Images were captured using a microscope (Leica, Wetzlar, Germany) and quantitatively analysed using ImageJ software.

### 2.3 Immunofluorescence (IF) Analysis

Fixed cells and paraffin sections were blocked with rapid blocking immunostaining buffer (P0260; Beyotime, China) for 15 min at room temperature for immunofluorescence staining. Samples were then incubated with primary antibodies overnight at 4°C. The cells were then labelled with Alexa Fluor 594 preabsorbed goat anti‐rabbit IgG (ab150084, 1:500, Abcam, UK) and Alexa Fluor 488 preabsorbed goat anti‐rabbit IgG (ab150077, 1:500, Abcam, UK) for 2 h at room temperature. Nuclei were stained with DAPI and observed using fluorescence microscopy.

### 2.4 Real‐Time Quantitative PCR (RT‐qPCR)

Total RNA was extracted from cells and tissues using TRIzol reagent (15596026, Invitrogen, USA), and cDNA was synthesized using the Gene Synthesis Kit (RR047Q, Takara, Japan), both according to the manufacturer's instructions. RT‐qPCR was performed using TB Green®PreMix Ex Taq™II (RR820A, Takara, Japan) and the StepOnePlus real‐time polymerase chain reaction system. GAPDH was used as an internal control. Gene expression was quantified using the 2‐ΔΔ Ct method. The primer sequences are listed in Table S2.

### 2.5 Western Blotting (WB)

Cells or muscle tissue were homogenized by adding phosphatase and protein hydrolase inhibitors (KGP2100; Keygen Biotech, China) to the RIPA lysis buffer. The protein concentrations were estimated and adjusted using the BCA method (ZJ102, Epizyme). Proteins were separated using SDS‐PAGE and transferred to PVDF membranes (Bio‐Rad, Hercules, CA, USA), which were blocked with 5% nonfat milk and then incubated with the primary antibody at 4°C overnight. Membranes were then incubated with the appropriate secondary antibody at 37°C for 1 h. The immunocomplexes were then visualized using Tanon™ High Signal ECL Protein Blotting Substrate (Tanon, Tanon, China) and an automated digital gel/chemiluminescence image analysis system (4600SF, Tanon, China). The antibodies used in this study are listed in Table S3.

### 2.6 Cell Proliferation and Differentiation

Mouse myoblast C2C12 cells were obtained from the Cell Bank of the Chinese Academy of Sciences (Shanghai, China) and cultured in DMEM (C11995500BT, Gibco, USA) supplemented with 10% foetal bovine serum FBS (Gibco, USA). All cells were cultured with 1% penicillin/streptomycin (Gibco, USA) at 37°C in 5% CO_2_. C2C12 myoblasts were maintained in a proliferative state for two days and then used for biochemical analyses. Mycoplasma contamination was not detected in any of the cell lines before use.

To stimulate differentiation, the cells were transferred to a differentiation medium (DMEM supplemented with 2% horse serum and 1% penicillin/streptomycin) at 70%–80% confluence. Myogenic differentiation assays were performed within seven days.

### 2.7 Lentiviral Transfection and Treat

Lentivirus constructs of keratocan‐overexpression (oe‐Kera), control‐overexpression (oe‐NC), keratocan‐shRNA (sh‐Kera) and control‐shRNA (sh‐NC) were purchased from Genecem (Shanghai, China). The sequences of keratocan‐targeting shRNAs and non‐targeting control shRNAs are shown in Supporting Information Table S4. In total, 5 × 10^4^ C2C12 cells were inoculated into 12‐well plates, and the virus and corresponding infection‐enhancing solution were added at 30%–50% confluency. After 24 h, the culture medium was replaced with fresh, complete medium. GFP expression was observed 3 days after infection. Puromycin (4 μg/mL; ST551‐10 mg, Beyotime, China) was applied for 10 days to screen the transfected cell lines. The transfection efficiency of the cells was determined using RT‐qPCR and western blotting.

Cells were treated with PI3K inhibitor (LY294002, 10 μM, 24 h; MCE) and PI3K activator (740Y‐P, 10 μM, 24 h, MCE). The inhibitors and activators were all dissolved in DMSO. The DMSO concentration in all experimental groups was consistently lower than 0.1%.

### 2.8 RNA Sequencing and Data Analysis

After induction and differentiation into mature myotubes, C2C12 cells transfected with oe‐NC or oe‐Kera were harvested and sent to Jikai for up‐sequencing on an Illumina Novaseq6000 sequencer. Differentially expressed genes (DEGs) were analysed using the DESeq2 package of R software; genes with *p* < 0.05 and|log_2_ (fold change)| ≥ 1 were defined as DEGs. The results were visualized using the ggplot2 package (version 3.3.6) to generate heat maps and volcanic plots.

### 2.9 Laboratory Animals

Five male C57/BL6J mice aged 3 and 24 months and 15 male SAMP8 senescent mice aged 8 months were obtained from the Experimental Animal Center of Fujian Provincial Hospital. All animal experiments were approved by the Ethics Committee for Animal Experiments of Fujian Provincial Hospital (IACUC‐FPH‐SL‐20240511[0264]), and all animals were cared for in accordance with the Guide for the Care and Use of Laboratory Animals.

### 2.10 Animal Experimental Design

Senescence‐accelerated mouse 8 (SAMP8) mice, a model of sarcopenia, were used in this study. These mice are considered to be in the early stages of sarcopenia at 8 months of age and develop a sarcopenic phenotype at 10 months of age [15, 16]. Fifteen 8‐month‐old male SAMP8 mice were randomly divided into three groups: control (*n* = 5), AAV9‐oe‐Scramble (*n* = 5) and AAV9‐oe‐Kera (*n* = 5). Control mice were injected with equal amounts of PBS, the AAV9‐oe‐Scramble group was injected with scramble oeRNA vector, and the AAV9‐oe‐Kera group was injected with AAV9 vector encoding oe‐Kera. All injections were into the gastrocnemius (GA) muscle at multiple sites. AAV9 particles were diluted in PBS, resulting in a final concentration of 2 × 10^11^ vector genome (VG)/GA, with a multipoint injection of 20 μL bilaterally. Eight weeks after the injection, all animals were placed on a treadmill for behavioural testing. All animals were euthanized and weighed, and the bilateral gastrocnemius muscles were sectioned, weighed, and subjected to histological analysis after removal.

### 2.11 Treadmill Exercise Test

Behavioural testing was performed 8 weeks after the AAV9 virus injection. Mice went through an acclimation period prior to formal testing. On the first day, the mice were exercised on a treadmill at 0 cm/s for 5 min, followed by 5 min at a slow speed of 10 cm/s. On the second day, mice were placed on the treadmill and gradually accelerated by 5 cm/s at 2‐min intervals on a 13% incline until they reached a level of exhaustion. If the animal's hind limbs remained on the treadmill for more than 10 s, the exhaustion criteria were met. At the end of the test, the machine automatically recorded the maximum speed, distance run, and time to exhaustion.

### 2.12 Hind Limb Grip Strength Assessment

The hind limb grip strength of the mice was measured using a grip dynamometer (Shanghai Xin Ruan, China) prior to performance. The same individual underwent three hind limb grip strength assessments with a recovery time of 1–2 min, and grip strength was quantified as the average of the three trials.

### 2.13 Statistical Analysis

All experiments were repeated three times, and the data were analysed using GraphPad Prism (9.0, Graph Software, USA). Image J software version 1.53 (Java, NIH, USA) was used to analyse fluorescence images and Western blot bands to quantify protein expression. Fisher's exact test was used to compare the sexes of the two groups. The Shapiro–Wilk test was used to test for normality. Experimental data are expressed as mean ± standard deviation (SD). Comparisons between univariate two‐group information were made using the Student's *t*‐test or the nonparametric Mann–Whitney test. Comparisons between multiple groups were performed using one‐way analysis of variance (ANOVA) with Tukey's post‐hoc test, and multiple comparisons of different groups with different outcomes were performed using parametric two‐way ANOVA. The independent variables included the different treatment methods (Control, AAV9‐oe‐Scramble, and AAV9‐oe‐Kera groups), while the dependent variables were maximum running speed, running distance, exhaustion time, hind limb muscle strength, and gastrocnemius cross‐sectional area (CSA). A *p*‐value < 0.05 was considered statistically significant.

## 3 Results

### 3.1 Screening of the Candidate Gene Keratocan

**FIGURE 1:**
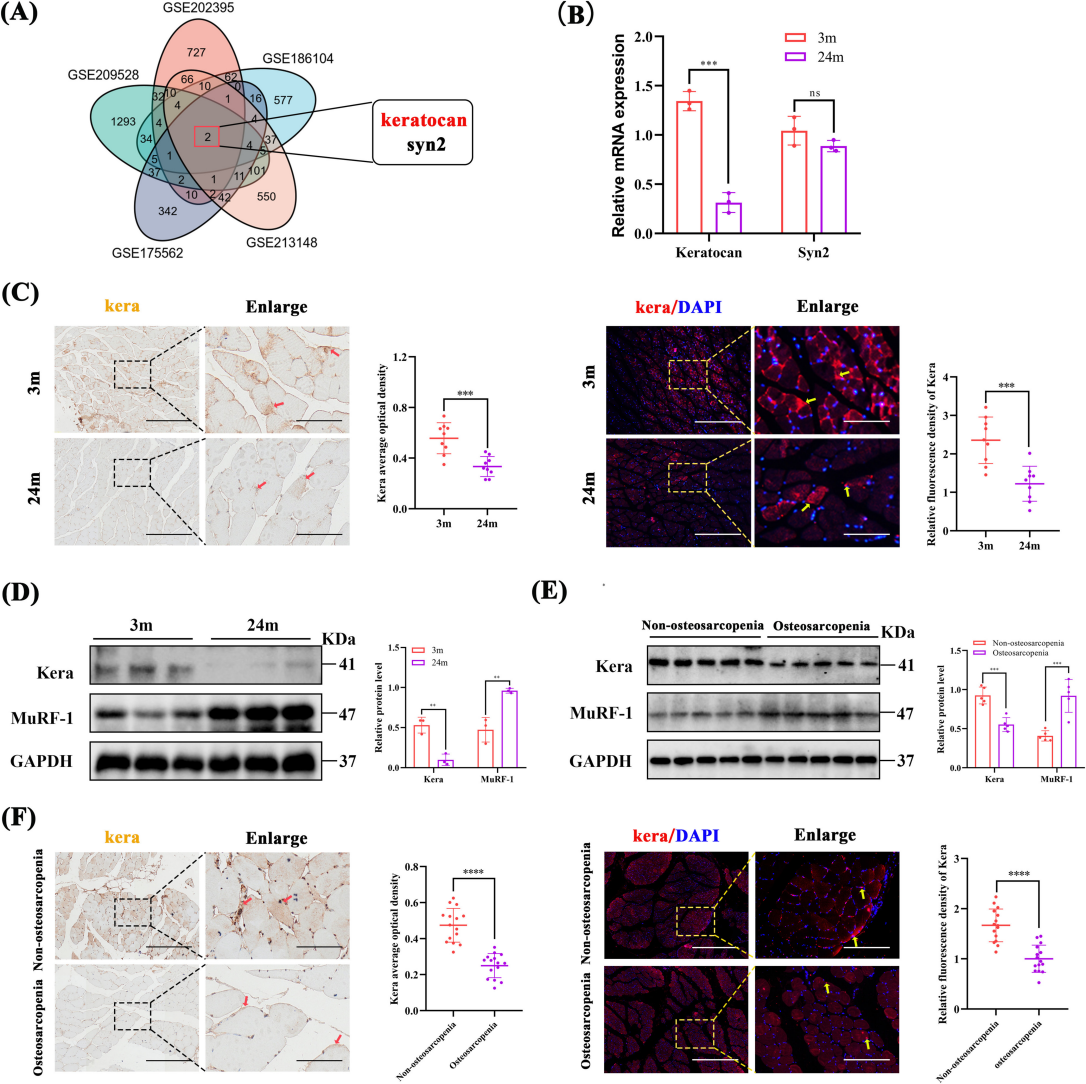
Keratocan is downregulated in the skeletal muscle of patients with osteosarcopenia and aged mice. (A) Venn diagram showing the overlap between differential mRNA expression in sarcopenia and non‐sarcopenia muscle and differential mRNA expression in osteoporotic and non‐osteoporotic bone. (B) Keratocan versus Syn2 mRNA levels in GAs of 3‐month‐old non‐osteosarcopenia and 24‐month‐old osteosarcopenia mice, *n* = 3. (C) Representative IHC and IF staining in 3‐month‐old non‐osteosarcopenia and 24‐month‐old osteosarcopenia mice, scale bar = 200 μm, the dashed line enlarges the area of the scale bar (50 μm). (D) Western blotting and quantitative analysis of keratocan and Murf‐1 protein expression in 3‐month‐old non‐osteosarcopenia and 24‐month‐old osteosarcopenia mice, *n* = 3. (E) Western blotting and quantitative analysis of Kera and MuRF‐1 protein expression, *n* = 5. (F) Representative IHC and IF staining in non‐osteosarcopenia and osteosarcopenia patients, scale bar = 200 μm, the dashed line enlarges the area of the scale bar (50 μm). (C) *n* = 3, three fields per sample were selected. (F) *n* = 5, three fields per sample were selected. For all statistical plots, values are expressed as mean ± SD. ns, *p* > 0.05, **p < 0.01, ***p < 0.001, ****p < 0.0001. Statistical significance was determined using Student's *t*‐test (for B, C, D, E, and F).

In conjunction with the high‐throughput GEO database, we analysed the DEGs in the muscles (GSE209528, GSE175562, and GSE213148) of non‐osteoporosis and osteoporosis mice, DEGs in non‐osteoporosis and osteoporosis mouse bones (GSE186104 and GSE202395), and selected the intersection to screen the middle two candidate genes (keratocan and Syn2; Figure 1A). We also examined the expression of keratocan and Syn2 in the muscles of osteosarcopenia mice. Laminin staining showed that 24‐month‐old mice developed significant muscle atrophy, as evidenced by a reduction in the cross‐sectional area of muscle fibres, compared with 3‐month‐old mice (Figure S1A). IF staining for slow and fast myosin heavy chain (MyHC) identified type I and type II fibres. One main feature of sarcopenia is the loss of fast muscle fibre types (IIx and IIa) [17]. IF staining showed a decrease in the proportion of type II muscle fibres and an increase in the proportion of type I muscle fibres in the skeletal muscles of 24‐month‐old mice compared to 3‐month‐old mice (Figure S1B). Assessment of hindlimb grip strength showed that 24‐month‐old mice had significantly reduced strength compared with 3‐month‐old mice (Figure S1C). In addition, 24‐month‐old mice had decreased BMD, increased BS/BV, decreased BS/TV, decreased BV/TV, decreased Tb. N, and decreased Tb. Th compared with 3‐month‐old mice (Figure S1D). These showed that the 24‐month‐old mice had significant muscle loss and osteoporosis. The expression of keratocan was significantly downregulated in the muscles of 24‐month‐old mice compared with 3‐month‐old mice (Figure 1B), whereas there was no significant difference in the expression level of Syn2 between the two groups. The association between keratocan and osteoporosis has been previously reported, but the association between keratocan and sarcopenia has not; therefore, we chose keratocan for our subsequent study on sarcopenia.

### 3.2 Keratocan Is Downregulated in Muscle During Age‐Associated Osteosarcopenia

We examined keratocan expression during osteosarcopenia progression to determine whether keratocan is involved in skeletal muscle development. The expression of E3 ubiquitin ligase (Murf‐1) is significantly upregulated in varying muscle atrophy conditions [18]. Western blotting (Figure 1D) revealed significant muscle atrophy in gastrocnemius (GA) muscle samples from 24‐month‐old mice, with significantly lower levels of keratocan than in younger muscles. Similar results were obtained using IF and IHC staining (Figure 1C).

A total of 10 muscle samples were collected from patients who underwent hip surgery in our orthopaedic department and categorized into non‐osteosarcopenia (*n* = 5) and osteosarcopenia (*n* = 5) groups. The relevant clinical data of all included cases are presented in Table S5. Laminin staining (Figure S2A) showed a significant decrease in CSA in the quadriceps femoris of patients with osteosarcopenia. IF staining of fast and slow MyHC (Figure S2B) showed that the proportion of type II muscle fibres decreased and that of type I muscle fibres increased in the skeletal muscles of patients with sarcopenia and osteoporosis. Keratocan protein levels were significantly lower in the skeletal muscles of patients with osteosarcopenia than those without osteosarcopenia (Figure 1E,F).

### 3.3 Keratocan is Upregulated During the Myogenic Differentiation of C2C12 Cells

The C2C12 cell line differentiates rapidly, forms contractile myotubes, produces characteristic myosins, and is widely used in medical research on muscle development [19]. The expression of keratocan was significantly higher in the differentiation medium for 7 days than in the growth medium (Figure S3A,B). Double immunofluorescence staining co‐localization after differentiation into myotubes (Figure S3C) showed that keratocan was highly expressed in fused myotubes.

### 3.4 Overexpression of Keratocan Promotes the Proliferation and Differentiation of C2C12 Myoblasts

**FIGURE 2:**
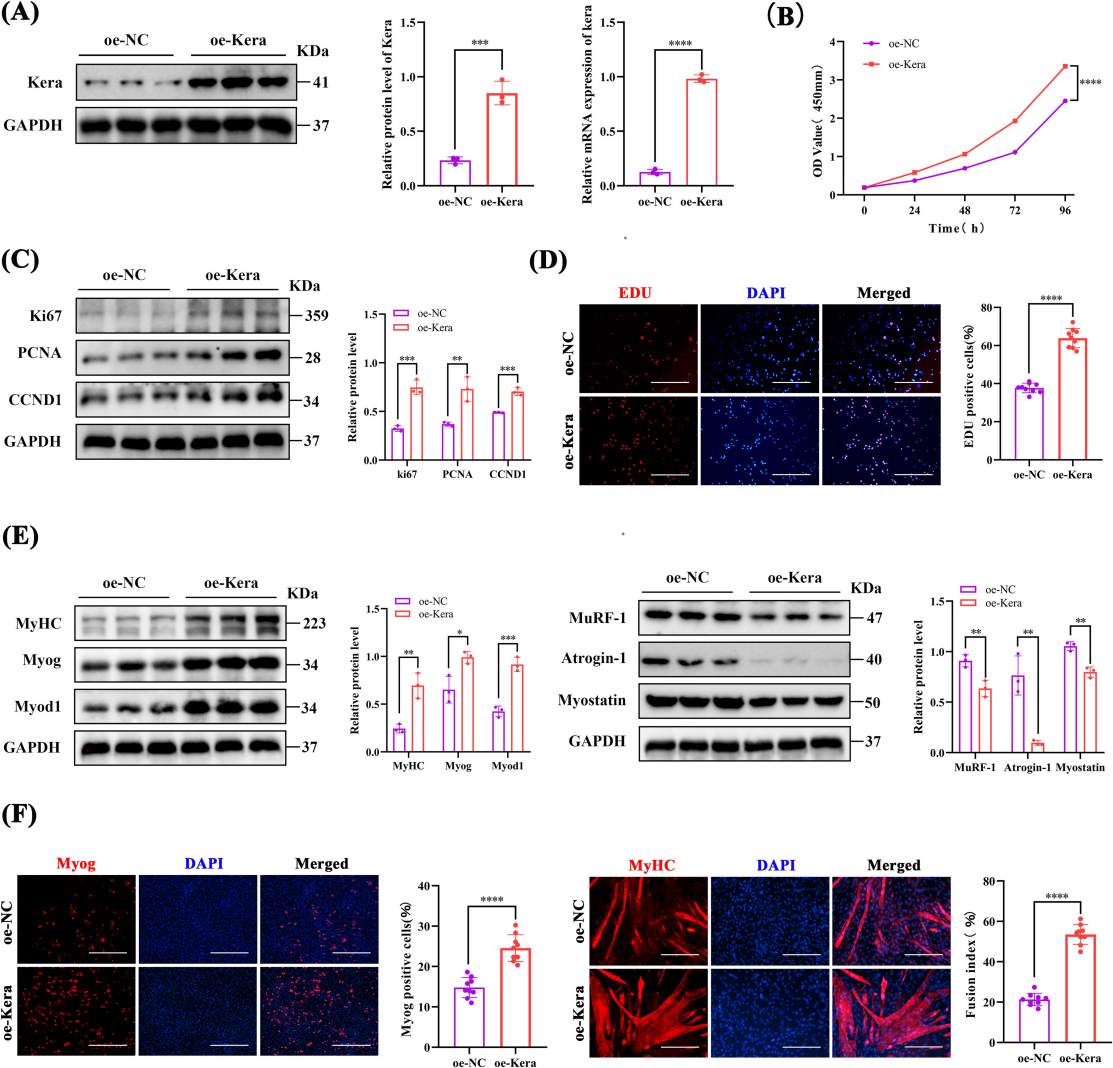
Overexpression of keratocan promotes the proliferation and differentiation potential of C2C12 myoblasts. (A) Keratocan mRNA and protein levels in oe‐NC and oe‐Kera after 48 h of transfection, *n* = 3. (B) Cell proliferation of oe‐NC and oe‐Kera was detected using the CCK8 method, *n* = 3. (C) Western blotting and quantitative analysis of ki67, PCNA, and CCND1 protein levels in oe‐NC and oe‐Kera, *n* = 3. (D) Representative EdU staining and positive cell count of oe‐NC and oe‐Kera, scale = 200 μm. (E) Western blotting and quantitative analysis of MyoG, MyoD1, MyHC, MuRF‐1, Atrogin‐1, and myostatin protein expression in oe‐NC and oe‐Kera, *n* = 3. (F) oe‐NC and oe‐Kera representing Myog staining patterns and proportion of MyoG‐positive cells, scale = 100 μm. The representative MyHC staining of oe‐NC and oe‐Kera and the quantitative evaluation of the myoduct fusion index, scale = 200 μm. (D, F) *n* = 3, three fields per sample were selected. For all statistical plots, values are expressed as mean ± SD. *p < 0.05, **p < 0.01, ***p < 0.001, ****p < 0.0001. Statistical significance was determined using Student's *t*‐test (for A, B, C, D, E and F).

The mRNA and protein levels of oe‐Kera were significantly higher than those of oe‐NC, indicating that keratocan was successfully overexpressed in C2C12 cells (Figure 2A). The CCK8 assay showed that oe‐Kera promoted cell proliferation (Figure 2B). Similarly, pro‐proliferative protein levels (Ki67, PCNA, and CCND1) were significantly elevated in oe‐Kera (Figure 2C). The proportion of EdU‐positive cells was elevated in oe‐Kera (Figure 2D). After induced differentiation, oe‐kera significantly increased the protein expression of myogenic differentiation marker genes, including myogenin (myoblast‐1), myoblast‐1 (Myod1) and MyHC (Figure 2E). In contrast, oe‐Kera significantly decreased the expression levels of genes associated with muscle atrophy, including E3 ubiquitin ligase (atrogin‐1 and Murf‐1) and myostatin. Keratocan overexpression may inhibit muscle atrophy by reducing muscle protein degradation through the ubiquitin‐proteasome pathway. IF staining of MyoG and MyHC (Figure 2F) showed that oe‐Kera significantly enhanced myogenic differentiation and myotube fusion.

### 3.5 Keratocan Deficiency Inhibits the Proliferation and Differentiation of C2C12 Myoblasts

**FIGURE 3:**
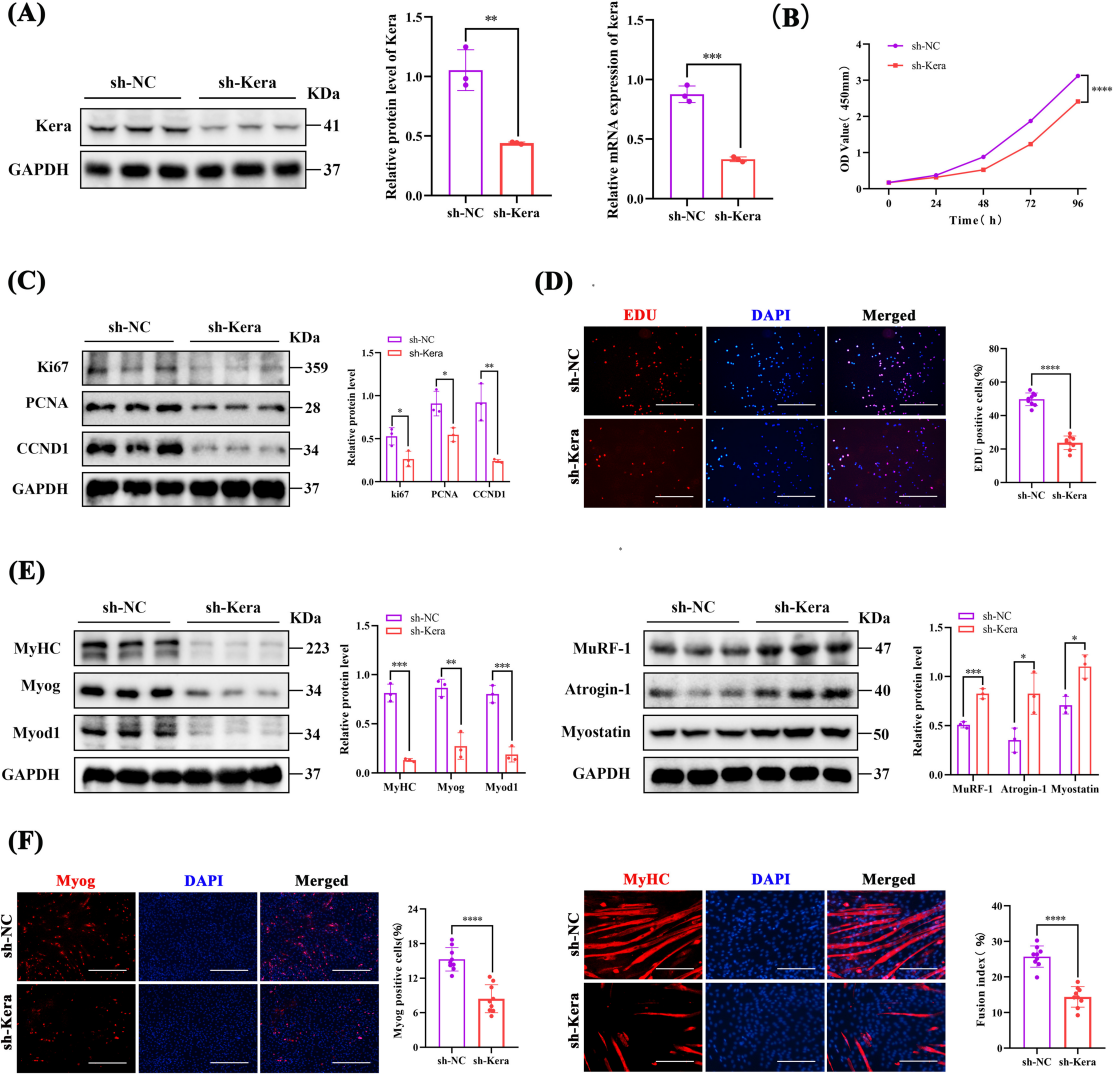
Keratocan knockdown inhibits the proliferation and differentiation potential of C2C12 myoblasts. (A) Keratocan mRNA and protein levels in sh‐NC and sh‐Kera after 48 h of transfection, *n* = 3. (B) CCK8 assay to detect cell proliferation in sh‐NC and sh‐Kera, *n* = 3. (C) Western blotting and quantitative analysis of ki67, PCNA, and CCND1 protein levels, *n* = 3. (D) Representative EdU staining and positive cell counts of sh‐NC and sh‐Kera, scale bar = 200 μm. (E) Western blotting and quantitative analysis of MyoG, MyoD1, MyHC, MuRF‐1, Atrogin‐1, and myostatin expression in sh‐NC and sh‐Kera, *n* = 3. (F) sh‐NC and sh‐Kera representing Myog staining patterns and proportion of MyoG‐positive cells, scale = 100 μm. The representative MyHC staining of sh‐NC and sh‐Kera and the quantitative evaluation of the myoduct fusion index, scale = 200 μm. (D, F) *n* = 3, three fields per sample were selected. For all statistical plots, values are expressed as mean ± SD. *p < 0.05, **p < 0.01, ***p < 0.001, ****p < 0.0001. Statistical significance was determined using Student's *t*‐test (for A, B, C, D, E, and F).

We confirmed that keratocan‐eGFP lentiviral vectors successfully knocked down keratocan in C2C12 cell lines (Figure 3A). The CCK8 assay showed that sh‐Kera inhibited cell proliferation (Figure 3B). Similarly, the protein expression of pro‐proliferative factors Ki67, PCNA and CCND1 were significantly reduced in sh‐Kera (Figure 3C). The EdU proliferation assay showed a decreased ratio of EdU‐positive cells in sh‐Kera (Figure 3D). After differentiation induction, sh‐Kera significantly decreased the expression levels of the marker genes for myogenic differentiation (Myog, Myod1 and MyHC), while the expression of the muscle atrophy‐related genes (Atrogin‐1, Murf‐1 and myostatin) was upregulated (Figure 3E). IF staining of Myog and MyHC showed that sh‐Kera inhibited myogenic differentiation and myotube fusion (Figure 3F).

### 3.6 RNA‐Seq Analysis of oe‐NC and oe‐Kera

**FIGURE 4:**
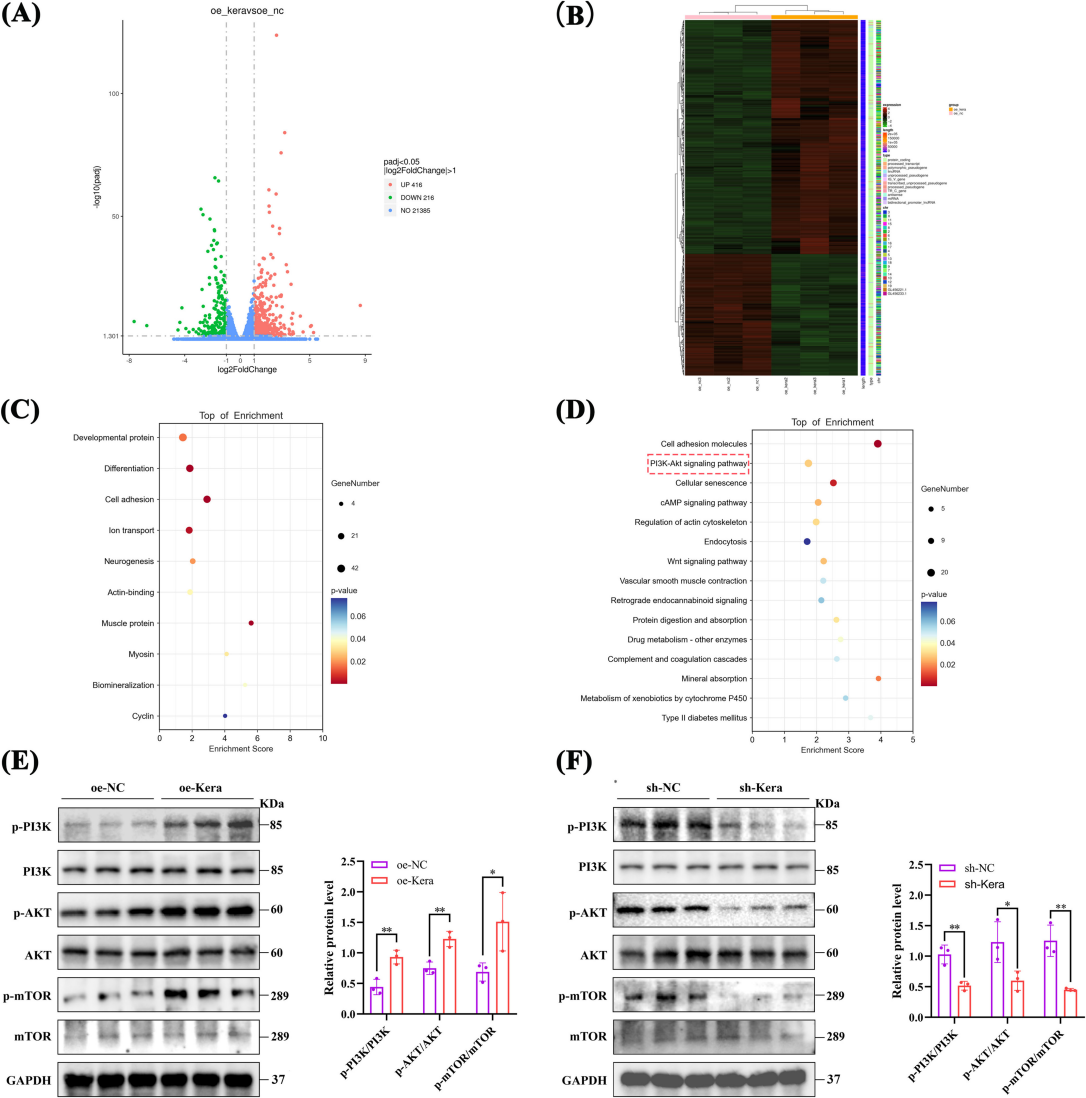
RNA‐seq analysis of oe‐NC and oe‐Kera (A) Volcano plots of RNA‐Seq data for C2C12 cells from the oe‐NC and oe‐Kera groups. Red and blue colours represent up‐ and downregulated genes, respectively. (B) Heatmap showing differentially expressed genes (DEGs). (C) GO enrichment analysis of DEGs. (D) KEGG enrichment analysis of DEGs. (E) Western blotting and quantitative analysis of p‐PI3K, p‐AKT, and p‐mTOR expression in oe‐NC and oe‐Kera, *n* = 3. (F) Western blotting and quantitative analysis of p‐PI3K, p‐AKT, and p‐mTOR expression in sh‐NC and sh‐Kera, *n* = 3. For all statistical plots, values are expressed as mean ± SD. *p < 0.05, **p < 0.01. Statistical significance was determined using Student's *t*‐test (for E and F).

A total of 632 DEGs were obtained, including 416 upregulated and 216 downregulated genes (Figure 4A,B). These DEGs were found to be involved in muscle protein synthesis and development, biomineralization, cell adhesion and cell differentiation (Figure 4C). KEGG pathway enrichment analysis showed that the pathway with the highest enrichment was the PI3K/AKT pathway (Figure 4D). The PI3K/AKT/mTOR pathway is a key pathway regulating the proliferation and differentiation of skeletal muscle satellite cells, as well as protein synthesis in myocytes [20]. The protein phosphorylation levels of PI3K, AKT and mTOR were upregulated in oe‐Kera compared with oe‐NC (Figure 4E) and downregulated in sh‐Kera compared to sh‐NC (Figure 4F).

### 3.7 Keratocan Regulates the Proliferation and Differentiation of C2C12 Myoblasts Through the PI3K/AKT/mTOR Signalling Pathway

**FIGURE 5:**
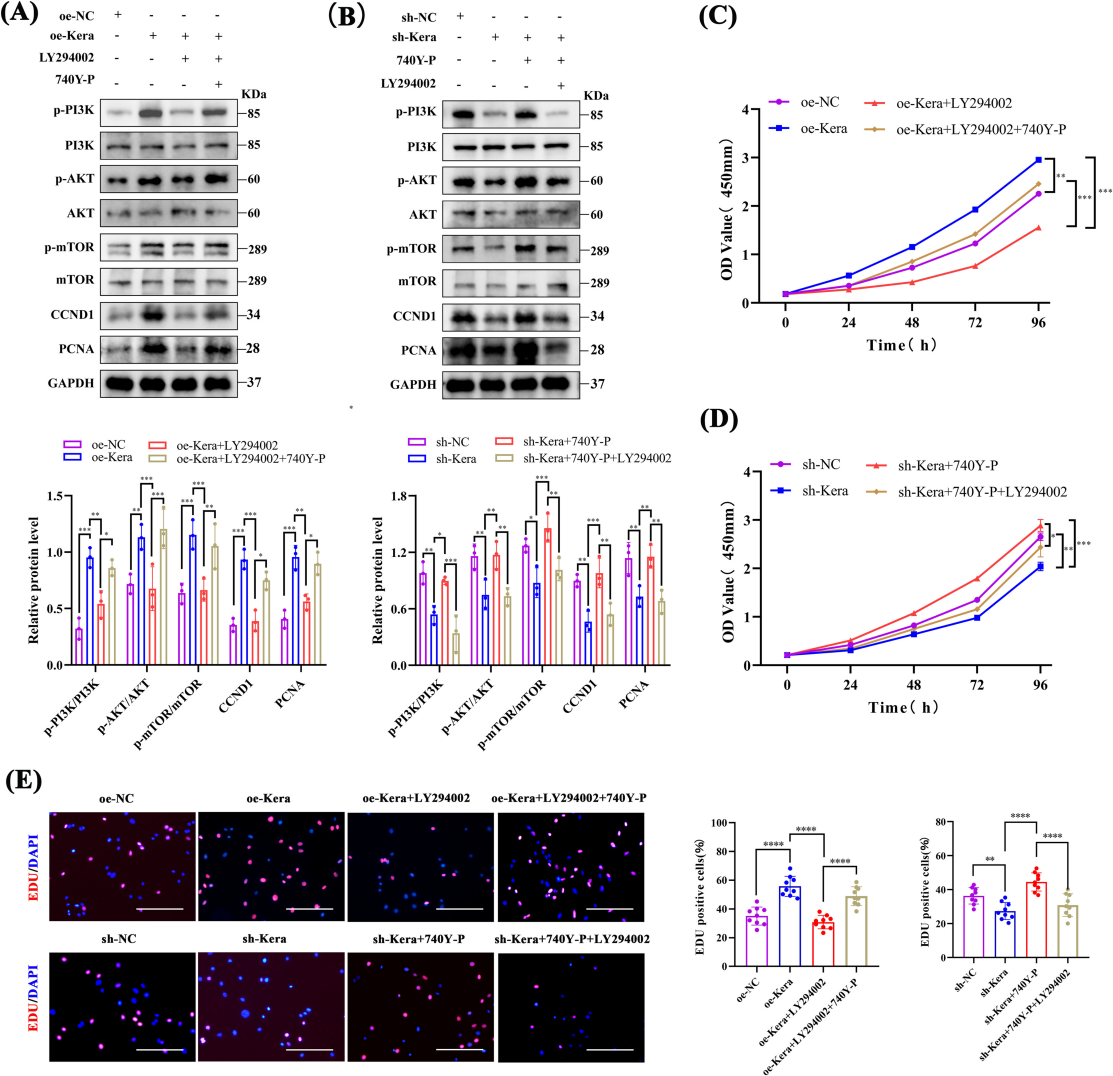
Keratocan regulates C2C12 myoblast proliferation through the PI3K/AKT/mTOR signalling pathway. (A) Western blotting and quantitative analysis of p‐PI3K, p‐AKT, p‐mTOR, CCND1, and PCNA protein expression in oe‐NC, oe‐Kera, oe‐Kera+LY294002, and oe‐Kera+LY294002 + 740Y‐P, *n* = 3. (B) Western blotting and quantitative analysis of p‐PI3K, p‐AKT, p‐mTOR, CCND1, and PCNA protein expression in sh‐NC, sh‐Kera, sh‐Kera+740Y‐P, and sh‐Kera+740Y‐P + LY294002, *n* = 3. (C) CCK8 assay for cell proliferation of oe‐NC, oe‐Kera, oe‐Kera+LY294002, and oe‐Kera+LY294002 + 740Y‐P, *n* = 3. (D) CCK8 assay for cell proliferation of sh‐NC, sh‐Kera, sh‐Kera+740Y‐P, and sh‐Kera+740Y‐P + LY294002, *n* = 3. (E) Representative EdU staining and positive cell counts for oe‐NC, oe‐Kera, oe‐Kera+LY294002, oe‐Kera+LY294002 + 740Y‐P, sh‐NC, sh‐Kera, sh‐Kera+740Y‐P, and sh‐Kera+740Y‐P + LY294002, scale bar = 100 μm; *n* = 3, three fields per sample were selected. For all statistical plots, values are expressed as mean ± SD. *p < 0.05, **p < 0.01, ***p < 0.001, ****p < 0.0001. One‐way ANOVA (for E) or two‐way ANOVA (for A, B, C and D).

LY294002 inhibited the phosphorylation of PI3K, AKT and mTOR and disrupted the promotion of myofibroblast proliferation‐associated proteins CCND1 and PCNA, while 740Y‐P promoted the phosphorylation of PI3K, AKT and mTOR in oe‐Kera (Figure 5A,B). Furthermore, CCK8 (Figure 5C,D) and EdU (Figure 5E) assays showed that LY294002 significantly inhibited oe‐Kera proliferation. 740Y‐P reversed the inhibitory effect of LY294002. Keratocan, like 740Y‐P, activates the PI3K/AKT/mTOR signalling pathway and promotes the proliferation of C2C12 myoblasts.

**FIGURE 6:**
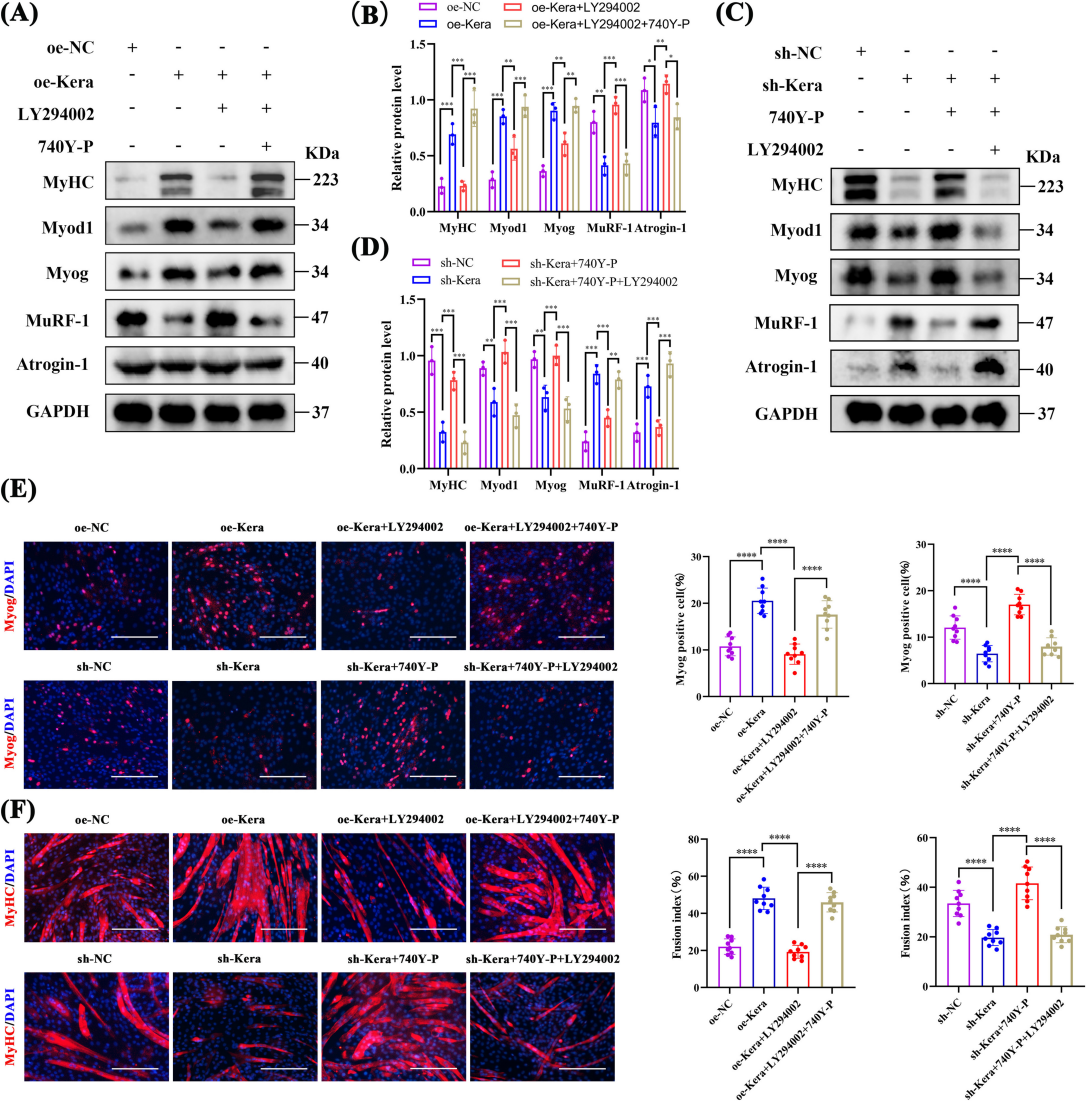
Keratocan regulates the differentiation potential of C2C12 cells through the PI3K/AKT/mTOR signalling pathway. (A, B) Western blotting and quantitative analysis of the protein expression of the myogenic markers MyoG, MyoD1, and MyHC, and the amyotrophic proteins Murf‐1 and Atrogin‐1 in oe‐NC, oe‐Kera, oe‐Kera+LY294002, and oe‐Kera+LY294002+740Y‐P, *n* = 3. (C, D) Western blotting and quantitative analysis of the protein expression of the myogenic markers MyoG, MyoD1, and MyHC, and the amyotrophic proteins MuRF‐1 and Atrogin‐1 in the sh‐NC, sh‐Kera, sh‐Kera+740Y‐P, and sh‐Kera+740Y‐P+LY294002 groups, *n* = 3. (E) Representative Myog staining plots and the percentage of MyoG‐positive cells of oe‐NC, oe‐Kera, oe‐Kera+LY294002, oe‐Kera+LY294002+740Y‐P, sh‐NC, sh‐Kera, sh‐Kera+740Y‐P, and sh‐Kera+740Y‐P+LY294002, scale = 200 μm. (F) Representative MyHC staining plots and muscle tubes for quantitative evaluation of the fusion index of oe‐NC, oe‐Kera, oe‐Kera+LY294002, oe‐Kera+LY294002+740Y‐P, sh‐NC, sh‐Kera, sh‐Kera+740Y‐P, and sh‐Kera+740Y‐P+LY294002, scale = 100 μm. (EF) *n* = 3, three fields per sample were selected. For all statistical plots, values are expressed as mean ± SD. *p < 0.05, **p < 0.01, ***p < 0.001, ****p < 0.0001. Statistical significance was determined using one‐way ANOVA (for E and F) or two‐way ANOVA (for B and D).

We then verified the role of the PI3K/AKT/mTOR pathway in regulating adult muscle differentiation in keratocytes. LY294002 suppressed the protein expression of the myogenic differentiation markers Myog, Myod1 and MyHC in oe‐Kera, while upregulating the expression of the muscle atrophy‐associated markers Atrogin‐1, Murf‐1 and myostatin. 740Y‐P reversed the inhibitory effects of LY294002 (Figure 6A,B). In contrast, 740Y‐P restored the protein expression of adult muscle differentiation markers Myog, Myod1 and MyHC, while downregulating the protein expression of muscle atrophy‐associated proteins Atrogin‐1, Murf‐1 and myostatin in sh‐Kera; the promoting function of 740Y‐P was disrupted after LY294002 treatment (Figure 6C,D). Similar results were obtained via the IF staining of Myog (Figure 6E) and MyHC (Figure 6F). Keratocan, like 740Y‐P, promotes myoblast differentiation by activating the PI3K/AKT/mTOR signalling pathway.

### 3.8 Keratocan Overexpression Improves Explosive Exercise Performance and Muscle Mass in SAMP8 Aging Mice

**FIGURE 7:**
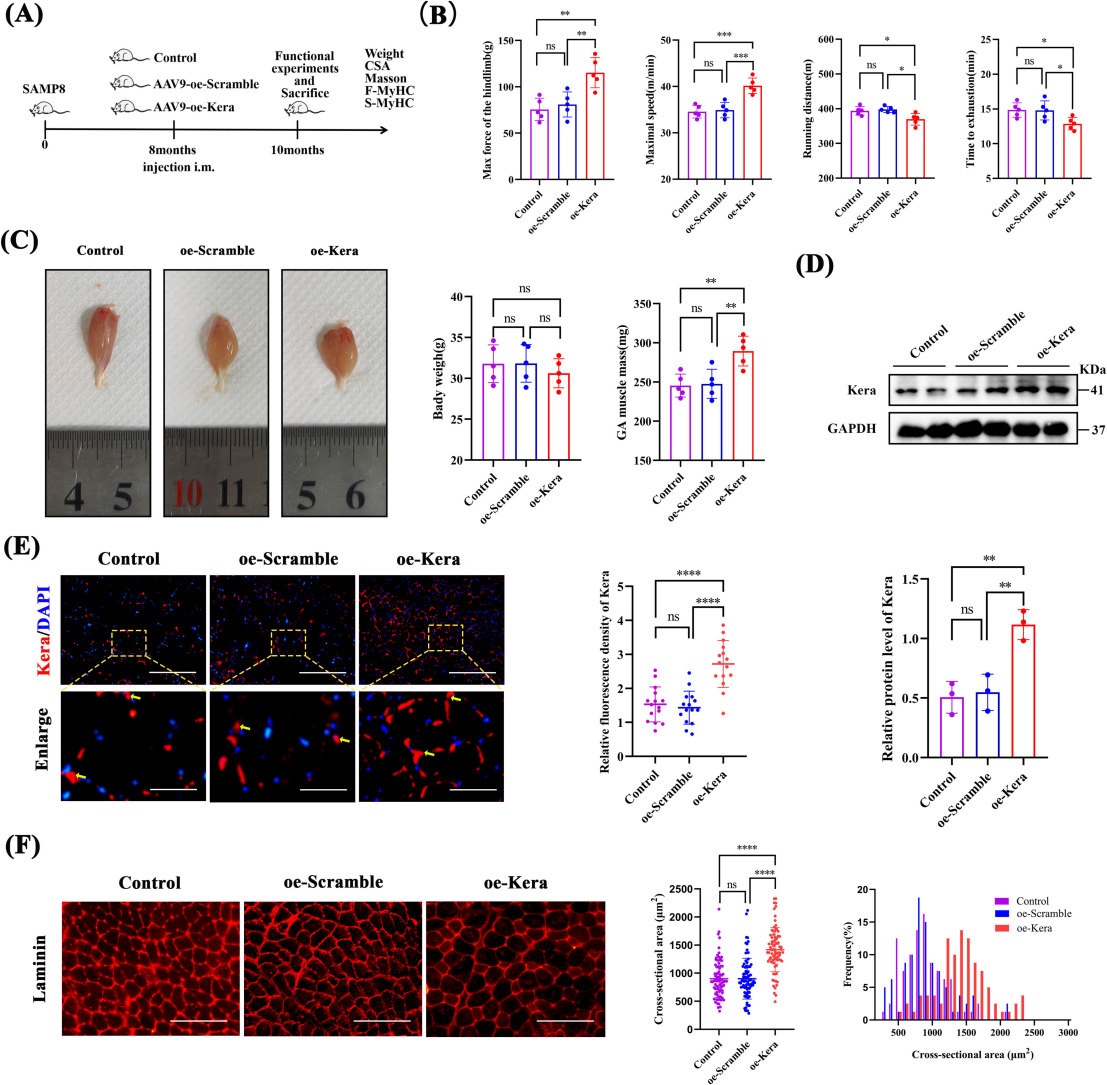
Overexpression of Keratocan improves exercise capacity and muscle mass in SAMP8 mice. (A) Schematic diagram of the animal experiments. (B) After two months of treatment, treadmill experiments were performed to assess maximum running distance, maximum running speed, and running time to fatigue, and grip strength experiments were performed to assess maximum hindlimb grip strength, *n* = 5. (C) Representative macroscopic photographs of GAs in each group, and quantitative analysis of body weights of mice and normalized weights of GAs at the time of sacrifice, *n* = 5. (D) Western blotting and quantitative analysis of keratocan protein expression in control, oe‐Scramble, and oe‐Kera groups, *n* = 3. (E) Representative IF staining of control, oe‐Scramble, and oe‐Kera, and the fluorescence intensity of keratocan. A magnified view of the yellow square regions is shown below, scale bar = 200 μm, the dashed line enlarges the area of the scale bar (50 μm); *n* = 5, three fields per sample were selected. (F) Representative Laminin staining for each group and quantitative analysis of muscle fibre CSA, scale = 200 μm; *n* = 5, 50 fibres per sample were selected. For all statistical plots, values are expressed as mean ± SD. ns, *p* > 0.05, *p < 0.05, **p < 0.01, ***p < 0.001, ****p < 0.0001. Statistical significance was determined using one‐way ANOVA (for B, C, D, E and F). GA, gastrocnemius; CSA, cross‐sectional area. Control, mice injected with the same volume of PBS; AAV9‐oe‐Scramble, mice injected with scrambled oeRNA vector control; AAV9‐oe‐Kera, mice injected with AAV9 vectors encoding oe‐Kera.

To validate the role of keratocan in vivo, we administered injections of the muscle‐forming specific promoter adeno‐associated virus AAV9‐oe‐Kera or control AAV9‐oe‐Scramble to 8‐month‐old SAMP8 mice (Figure 7A). We evaluated the locomotor ability, which was determined via the maximum grip strength of the hind limb, and performed the treadmill test (Figure 7B). The maximum grip strength and running speed of the hind limbs of oe‐Kera mice were significantly higher than those of the control group. Mice in the oe‐Kera group had reduced running distances and times to exhaustion compared to the control group. This indicates that keratocan improves explosive exercise performance but decreases endurance exercise performance. The oe‐kera‐treated group showed an increased mass of the gastrocnemius muscle, but body weight was unaffected compared with the control group (Figure 7C). There was a significant increase in the protein expression of keratocan in the gastrocnemius muscle of the oe‐Kera group, confirming good transduction efficiency of the AAV9 vector (Figure 7D,E). Laminin staining (Figure 7F) showed that the myofibre cross‐sectional area (CSA) and intermuscular fibrosis remained higher in the oe‐Kera group than in the control group.

### 3.9 Keratocan Overexpression Ameliorates Skeletal Muscle Atrophy and Promotes Muscle Development and Fast‐Twitch Fibre Synthesis in SAMP8 Aging Mice Through the PI3K/AKT/mTOR Signalling Pathway

**FIGURE 8:**
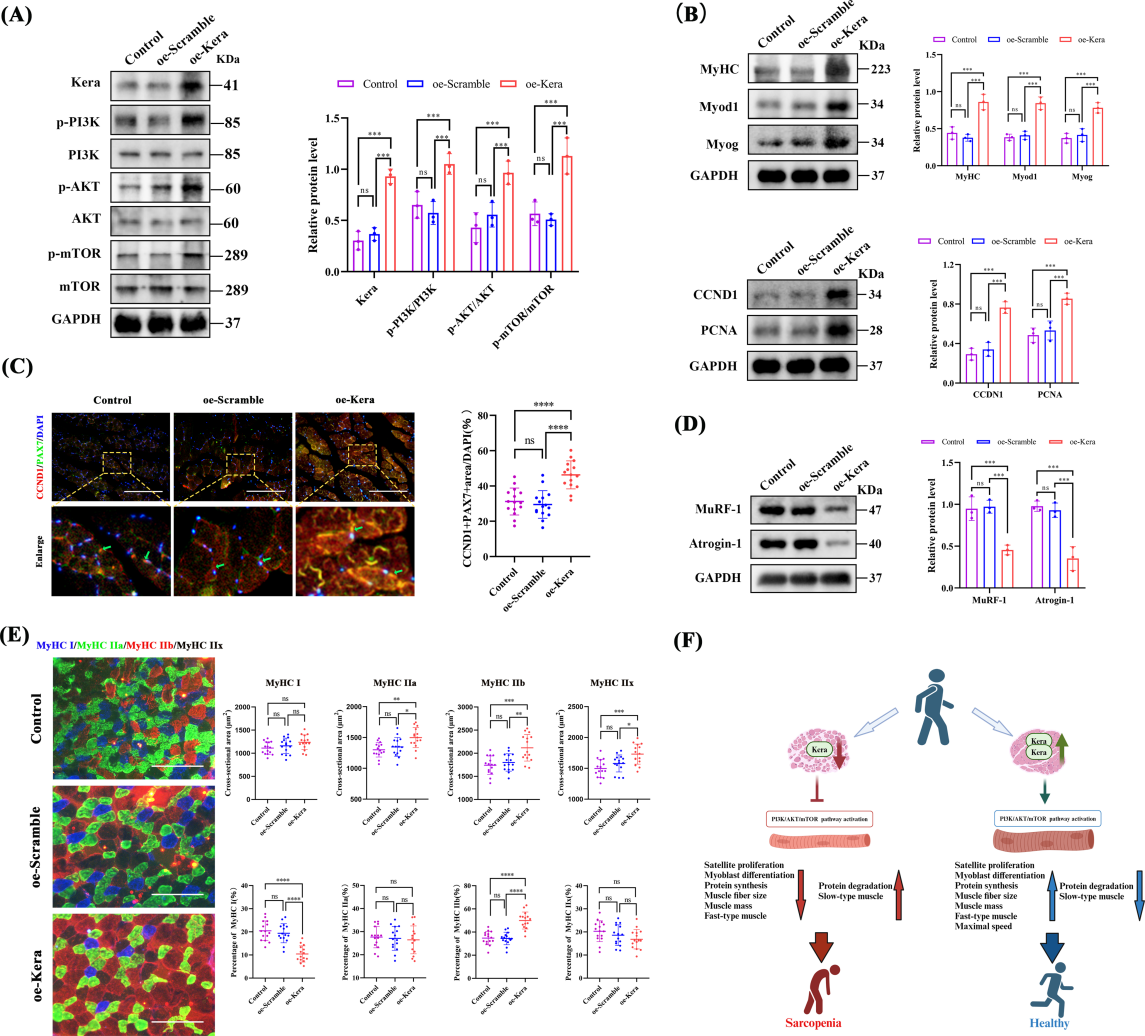
Keratocan overexpression promotes skeletal muscle development and rapid myofibre synthesis in SAMP8 mice via the PI3K/AKT/mTOR signalling pathway. (A) Western blotting and quantitative analysis of the protein expression of p‐PI3K, p‐AKT, and p‐mTOR in control, oe‐Scramble, and oe‐Kera, *n* = 3. (B) Western blotting and quantitative analysis of the protein expression of Myog, MyoD1, MyHC, CCND1, and PCNA in control, oe‐Scramble, and oe‐Kera, *n* = 3. (C) Representative IF staining and quantitative analysis of overlapping fluorescence of CCND1 and PAX7 in control, oe‐Scramble, and oe‐Kera GA muscles. The lower panel is an enlarged view of the area in the upper panel, indicated by yellow squares. Scale bar = 200 μm, the dashed line enlarges the area of the scale bar (50 μm). (D) Western blotting and quantitative analysis of the expression of amyotrophic proteins MuRF‐1 and Atrogin‐1 in control, oe‐Scramble, and oe‐Kera, *n* = 3. (E) Representative IF staining of different myofibre types, and quantitative analysis of CSA and percentage of different myofibrillar types of GA muscle. Scale bar = 100 μm. (F) Schematic summary of the regulatory role of Keratocan in myogenic differentiation. (C, E) *n* = 5, three fields per sample were selected. For all statistical plots, values are expressed as mean ± SD. ns, *p* > 0.05, *p < 0.05, **p < 0.01, ***p < 0.001, ****p < 0.0001. Statistical significance was determined using one‐way ANOVA (for C and E) or two‐way ANOVA (for A, B, and D). GA, gastrocnemius; CSA, cross‐sectional area. Control, mice injected with the same volume of PBS; AAV9‐oe‐Scramble, mice injected with scrambled oeRNA vector control; AAV9‐oe‐Kera, mice injected with AAV9 vectors encoding oe‐Kera.

AAV9‐oe‐Kera activated the PI3K/AKT/mTOR pathway more than the control and AAV9‐oe‐Scramble, as evidenced by a significant increase in the phosphorylation levels of PI3K, AKT and mTOR (Figure 8A). The oe‐Kera promoted the expression of the myofibre regenerative capacity markers Myog, Myod1 and MyHC (Figure 8B). Keratocan overexpression promoted the proliferation of muscle satellite cells in SAMP8 mice, as evidenced by the altered CCND1 and Ki67 expression (Figure 8B,C). oe‐Kera also significantly decreased the expression of muscle atrophy‐related proteins Atrogin‐1 and MuRF‐1 (Figure 8D), suggesting that the overexpression of keratocan has the potential to promote muscle development and slow down muscle atrophy in SAMP8 mice. IF staining of muscle fibre types (Figure 8E) showed that the oe‐Kera group had a significantly increased percentage of fast‐twitch muscle fibres encoding MyHC type IIb compared with the control and AAV9‐oe‐scramble groups. In contrast, the percentage of the slow‐twitch myosin isoform MyHC1 decreased in the oe‐Kera group, suggesting that keratocan promotes the transition from slow‐to‐fast muscle fibres. To assess the status of muscle fibre metabolism in vivo, we analysed SDH staining associated with muscle fibre oxidation (Figure S4). The results showed that Keratocan overexpression reduced the accumulation of metabolically active SDH‐positive fibres.

## 4 Discussion

Previous studies have reported that keratocan plays a role in regulating cellular processes and promoting the differentiation of bone progenitor cell lineages [14]. We found that keratocan expression was reduced in the bones of osteoporotic mice and in the muscles of sarcopenic mice, which is consistent with the results of previous studies; however, the role of keratocan in sarcopenia has not yet been reported. We aimed to determine if keratocan could ameliorate sarcopenia by promoting the proliferation and myogenic differentiation of myosatellite cells, thus providing a new therapeutic target for osteosarcopenia. The findings of this study are depicted in Figure 8F. We found that keratocan expression was significantly reduced in the skeletal muscle of aged osteosarcopenia mice and patients. Our in vitro experiments showed that keratocan was highly expressed during the differentiation of C2C12 myoblasts and participated in myotube fusion, suggesting that keratocan may promote the differentiation of C2C12 cells. We tested this hypothesis using lentiviral overexpression or knockdown of keratocan in C2C12 cells. Keratocan overexpression promoted proliferation and myogenic differentiation of myosatellite cells, whereas keratocan knockdown inhibited these processes. Mechanistic studies revealed that keratocan acts by activating the PI3K/AKT/mTOR signalling pathway, and in vivo experiments showed that keratocan overexpression in SAMP8 aging mice not only improved locomotor performance and increased muscle mass but also promoted skeletal muscle development by facilitating the proliferation and differentiation of muscle satellite cells, ameliorating skeletal muscle atrophy, and facilitating the synthesis of fast‐twitch muscle fibre types.

The PI3K/AKT/mTOR pathway, a key signalling pathway regulating the proliferation and differentiation of skeletal muscle satellite cells, is pivotal in regulating protein synthesis in myocytes [21, 22, 23]. In sarcopenia, the PI3K/AKT/mTOR pathway activity is usually decreased, leading to decreased muscle satellite cell proliferation, differentiation, and protein synthesis, resulting in decreased muscle mass and function [24]. First, PI3K activation promotes AKT phosphorylation, which activates downstream mTOR, thereby stimulating protein synthesis and promoting muscle cell proliferation [25]. AKT also inhibits the expression of muscle atrophy‐associated proteins Atrogin‐1 and Murf‐1 and reduces muscle protein degradation [26]. Second, mTOR can promote the expression of myogenic differentiation marker proteins, such as MyoD and Myog, to induce adult muscle differentiation [27]. Therefore, PI3K/AKT/mTOR pathway regulation may be a potential target for the treatment of sarcopenia. Keratocan is a leucine‐rich proteoglycan mainly localized to the extracellular matrix (ECM) and plays an important role in maintaining the extracellular environment and cellular energy. In our RNA sequencing analysis, many differential genes associated with the PI3K/AKT pathway are extracellular matrix components. As shown in the PI3K/AKT pathway diagram (Figure S5), these increases in the extracellular matrix may activate the PI3K/AKT pathway by regulating the cellular microenvironment. We found that keratocan, an important regulatory mediator of P13K/Akt/mTOR signalling, promotes the proliferation and myogenic differentiation of C2C12 cells via this pathway. In vivo experiments showed that keratocan overexpression upregulated phosphorylation of the PI3K/Akt/mTOR pathway, promoting muscle development and attenuating the development of muscle atrophy.

Muscle atrophy is associated with proteolytic metabolism. Previous studies have shown that the expression of two key ubiquitin ligases, Murf‐1 and Atrogin‐1, in the protein degradation signalling pathway significantly increases during skeletal muscle atrophy [28]. Activated Murf‐1 and Atrogin‐1 lead to increased protein ubiquitination and accelerated proteolysis, resulting in increased muscle wasting. This muscle atrophy signalling pathway is inhibited by PI3K/Akt/mTOR pathway activation [29]. Here, these two genes were used as markers to determine the effects of keratocan knockdown or overexpression on protein hydrolysis. The in vitro overexpression of keratocan drastically reduced the expression of Murf‐1 and Atrogin‐1, and the degree of pathway activation was regulated by LY294002 or 740Y‐P. We observed that keratocan overexpression enhanced myofibre morphological parameters and muscle strength and downregulated the expression levels of Murf‐1 and Atrogin‐1 in SAMP8 mice. Keratocan reduced skeletal muscle protein hydrolysis and enhanced protein deposition via the PI3K/Akt/mTOR pathway.

Skeletal muscle contains different types of myofibres. Mature skeletal muscle fibres are classified as slow oxidative (MyHC I), fast oxidative (MyHC IIa), fast glycolytic (MyHC IIb), or intermediate (MyHC IIx) based on different MyHC isoforms [30] (S1, S2). Under the influence of certain factors, different myofibre types can be converted to each other to adapt or respond to physiological or pathological changes in the body. Myofibre‐type conversion is directly related to several human muscular and metabolic diseases, such as muscle wasting, which results in increased slow muscle fibres and decreased fast muscle fibres [31]. Chronic liver disease results in the transformation of myofibres from type IIb to type I [32]. Changes in the composition of different myofibre types occur as differentiation proceeds [33]. Aging, including sarcopenia, is associated with a decreased number of myofibres, as well as a decreased size of type II, but not type I, fibres [34]. Atrophy of fast fibres occurs more markedly with age; the loss of skeletal muscle mass is largely attributed to the reduced size of type II fibres [35]. Here, keratocan overexpression promoted development, dominated mainly by type II muscle fibres, along with a shift in fibre type from type I (oxidative) slow muscle fibres to larger type IIb (glycolytic) fast muscle fibres. The increase in fast skeletal muscle fibres improved explosive exercise performance in mice, as confirmed by maximal running speed and hind limb muscle strength tests, while the decrease in slow skeletal muscle fibres reduced the endurance of mice, as confirmed by the treadmill fatigue test.

In skeletal muscle, metabolic switching is often associated with changes in myofibre type [S3–5]. However, a study by Pereyra [S6] and colleagues found that skeletal muscle can undergo a metabolic switch in fibre type without altering myosin heavy chain expression. This finding suggests that metabolic switching is not always accompanied by changes in fibre type, reflecting the complexity of skeletal muscle in between. Our preliminary results suggest that Keratocan mediates changes in the structural phenotype of myosin, as reflected in the staining of fast and slow muscle fibres. Interestingly, we also found that Keratocan affected the metabolic pattern of muscle fibres, mainly by reducing the oxidative phosphorylation metabolic phenotype, which is consistent with the trend of our fibre types. Therefore, we speculate that Keratocan proteins not only drive structural phenotypic changes but may also be involved in metabolic transformation.

The transformation of muscle fibre types is influenced by numerous signalling pathways and regulatory factors. Studies have reported that the homeostatic activation of AKT increases fast/glycosylated fibres, promotes muscle hypertrophy and strength enhancement and that AKT signalling may act as a mediator of type II muscle hypertrophy [36, 37]. A recent study reported that phosphoglycerate translocase 2 activates the PI3K/AKT signalling pathway and inhibits FOXO1 phosphorylation, which in turn inhibits mitochondrial function and promotes fast‐twitch myofibril formation [38]. Similarly, this experiment demonstrated that keratocan promotes rapid myofibrillar fibrillogenesis via the PI3K/Akt/mTOR pathway. Other regulatory factors and signalling pathways may be involved in this process; the specific mechanisms must be explored in depth.

This study had several limitations. First, the in vivo experiments focused only on keratocan overexpression without evaluating the effect of keratocan knockdown. Future animal studies evaluating gain‐ and loss‐of‐function models may help better elucidate the mechanisms of keratocan‐mediated sarcopenia progression. Second, using SAMP8 mice as an animal model introduced some limitations. SAMP8 mice are the most commonly used accelerated aging mouse model in SP studies [39]; however, they may not represent sarcopenia caused by natural aging processes. In the future, other animal aging models should be used for further verification.

In conclusion, our results suggest that the overexpression of keratocan has beneficial effects on the proliferation and differentiation of myosatellite cells and that keratocan may ameliorate muscle atrophy through the PI3K/AKT/mTOR signalling pathway, promoting skeletal muscle development, increasing fast muscle fibre synthesis, and improving muscle function. This study identified keratocan as a biological target for potentially treating sarcopenia, providing an important basis for developing new targeted drugs for treating osteosarcopenia. This should be the focus of future research.

## Ethics Statement

All animal experiments were approved by the Animal Ethics Committee of Fujian Provincial Hospital (IACUC‐FPH‐SL‐20240511[0264]). Besides, this study was performed in line with the principles of the Declaration of Helsinki. Approval was granted by the Ethics Committee of Fujian Provincial Hospital (No. K2023–06‐008). Informed consent was obtained from individual participants and/or their legal guardians in the study. Besides, the accessed patient data complied with relevant protection, privacy guidelines and regulations.

## Consent

Not applicable.

## Conflicts of Interest

The authors declare no conflicts of interest.

## Supporting information


**Figure S1** Muscle atrophy and osteoporosis are significant in 24‐month‐old mice. (A) Representative Laminin staining of GA muscle and quantitative analysis of CSA in 3‐month‐old (left) and 24‐month‐old (right) mice, scale bar = 200 μm; *n* = 5, 50 fibres per sample were selected. (B) Representative IF staining of fast MyHC (top) or slow MyHC (bottom) in 3‐month‐old (left) and 24‐month‐old (right) mice, and quantitative analysis of the percentage, scale bar = 200 μm; *n* = 5, three fields per sample were selected. (C) Grip strength experiments to assess maximal hind limb grip strength, *n* = 5. (D) Representative μ‐CT and quantitative analysis of BMD, BV/TV, BS/TV, Tb. N, Tb. Th and Tb. Sp of the femurs of 3‐month‐old (left) and 24‐month‐old (right) mice, *n* = 5. For all statistical plots, values are expressed as mean ± SD. **p < 0.01, ***p < 0.001, ****p < 0.0001. Statistical significance was determined using Students *t*‐test (for A, B, C and D). Abbreviations: GA, gastrocnemius; CSA, cross‐sectional area.

**Figure S2** Muscle wasting is evident in patients with osteosarcopenia. (A) Representative Laminin staining and quantitative analysis of CSA in the quadriceps muscle in non‐osteosarcopenia (left) and osteosarcopenia (right) conditions, scale bar = 200 μm; *n* = 5, 50 fibres per sample were selected. (B) Representative IF staining for fast MyHC in non‐osteosarcopenia and osteosarcopenia (top) or slow MyHC (bottom) and quantitative analysis of the percentages, scale bar = 200 μm. (B) *n* = 5, three fields per sample were selected. For all statistical plots, values are expressed as mean ± SD. **p < 0.01, ****p < 0.0001. Statistical significance was determined using Students *t*‐test (for A, B and C). Abbreviation: CSA, cross‐sectional area.

**Figure S3** Keratocan is upregulated during C2C12 myoblast differentiation. (A) Western blotting and quantitative analysis of keratocan protein expression levels in GM and DM groups, *n* = 3. (B) Representative IF staining and fluorescence intensity of keratocan in GM and DM groups; scale bar = 200 μm, *n* = 5, three fields per sample were selected. (C) Representative IF staining of overlapping fluorescence of keratocan and MyHC four days after differentiation. For all statistical plots, values are expressed as mean ± SD. ** *p* < 0.01, **** *p* < 0.0001. Statistical significance was determined using Students *t*‐test (for A and B). Abbreviations: GM, growth medium; DM, differentiation medium.

**Figure S4** Representative SDH staining in GA muscle of each group and quantification of SDH‐positive fibres, scale bar = 100 μm. *n* = 5, three fields per sample were selected. **p < 0.01, ***p < 0.001. Statistical significance was determined using one‐way ANOVA. Abbreviations: GA, gastrocnemius. Control, mice injected with the same volume of PBS; AAV9‐oe‐Scramble, mice injected with scrambled oeRNA vector control; AAV9‐oe‐Kera, mice injected with AAV9 vectors encoding oe‐Kera.

**Figure S5** Diagram of the PI3K/AKT signalling pathway.

**Table S1** Summary of Mouse Sample Characteristics.
Table S2. Primers used in RT‐qPCR experiments.
Table S3. Antibodies and their application.
Table S4. The sequences of sh‐RNAs.
Table S5. Clinical characteristics of patients with or without Osteosarcopenia.

## Data Availability

The paper and the Supplementary Materials present all data needed to evaluate the conclusions. Datasets used and/or analysed during the current study are available from the corresponding author on reasonable request.
