# Supplementary figures and images for: Keratocan Improves Muscle Wasting in Sarcopenia by Promoting Skeletal Muscle Development and Fast‐Twitch Fibre Synthesis
Source: J Cachexia Sarcopenia Muscle. 2025 Feb 17;16(1):e13724. doi: 10.1002/jcsm.13724 (PMC11832428; doi:10.1002/jcsm.13724)

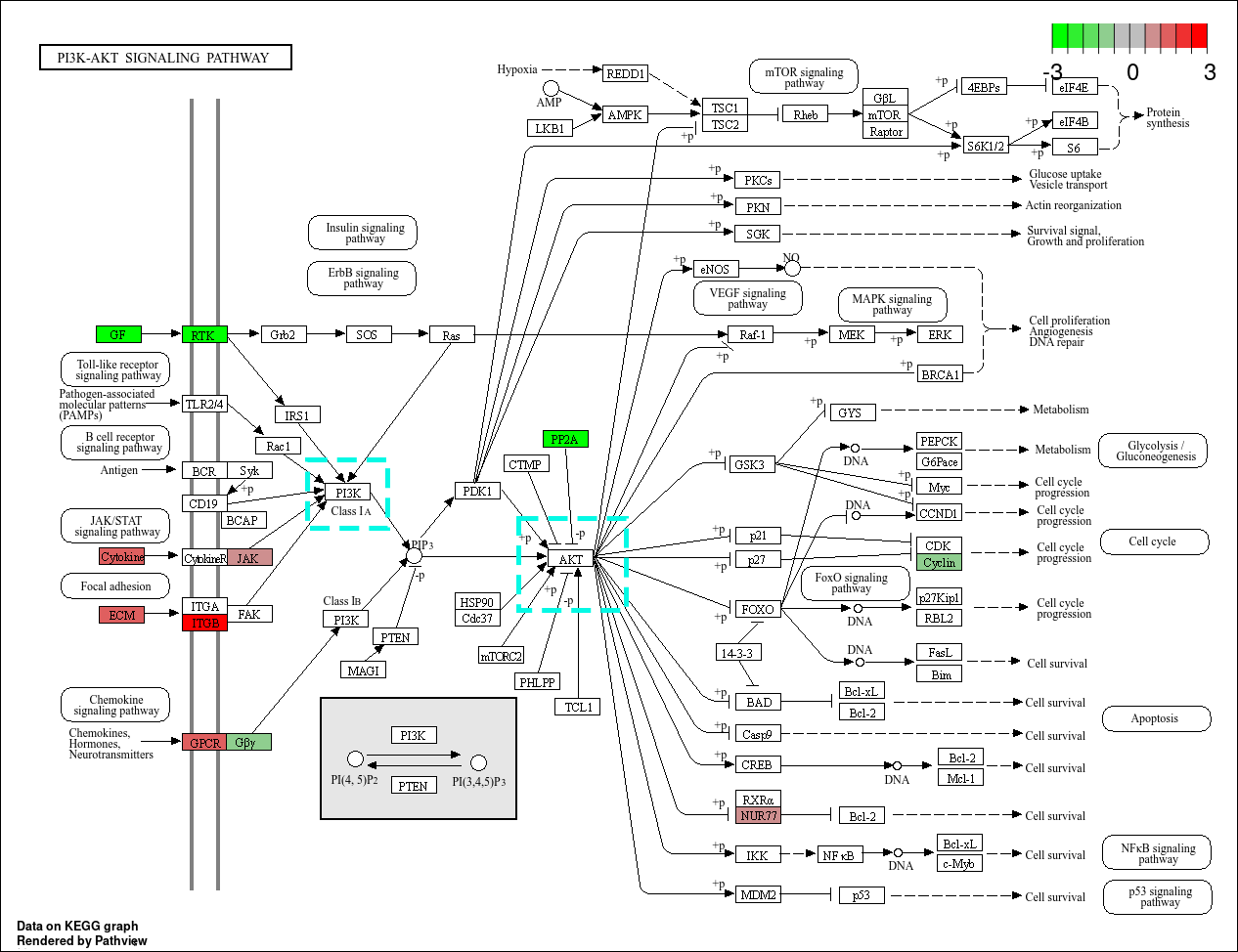

Supplement: Supplementary file 5 — Figure S5 Diagram of the PI3K/AKT signalling pathway. [file JCSM-16-e13724-s001.tif]
