# Supplementary material for: Keratocan Improves Muscle Wasting in Sarcopenia by Promoting Skeletal Muscle Development and Fast‐Twitch Fibre Synthesis
Source: J Cachexia Sarcopenia Muscle. 2025 Feb 17;16(1):e13724. doi: 10.1002/jcsm.13724 (PMC11832428; doi:10.1002/jcsm.13724)
Supplement: Supplementary file 6 — Table S1 Summary of Mouse Sample Characteristics. Table S2. Primers used in RT‐qPCR experiments. Table S3. Antibodies and their application. Table S4. The sequences of sh‐RNAs. Table S5. Clinical characteristics of patients with or without Osteosarcopenia. [file JCSM-16-e13724-s004.docx]

**Supplementary Material**

**Supplementary Figure 1. Muscle atrophy and osteoporosis are significant in 24-month-old mice.** (A) Representative **Laminin** staining of GA muscle and quantitative analysis of CSA in 3-month-old (left) and 24-month-old (right) mice, scale bar = 200 μm; n = 5, 50 fibers per sample were selected. (B) Representative IF staining of fast MyHC (top) or slow MyHC (bottom) in 3-month-old (left) and 24-month-old (right) mice, and quantitative analysis of the percentage, scale bar = 200 μm; n = 5, three fields per sample were selected. (C) Grip strength experiments to assess maximal hind limb grip strength, n = 5. (D) Representative μ-CT and quantitative analysis of BMD, BV/TV, BS/TV, Tb.N, Tb.Th, and Tb.Sp of the femurs of 3-month-old (left) and 24-month-old (right) mice, n = 5. For all statistical plots, values are expressed as mean ± SD. ***P* < 0.01, ****P* < 0.001, *****P* < 0.0001. Statistical significance was determined using Student's t-test (for A, B, C, and D). GA, gastrocnemius; CSA, cross-sectional area

**Supplementary Figure 2. Muscle wasting is evident in patients with osteosarcopenia.** (A) Representative **Laminin** staining and quantitative analysis of CSA in the quadriceps muscle in non-osteosarcopenia (left) and osteosarcopenia (right) conditions, scale bar = 200 μm; n = 5, 50 fibers per sample were selected. (B) Representative IF staining for fast MyHC in non-osteosarcopenia and osteosarcopenia (top) or slow MyHC (bottom) and quantitative analysis of the percentages, scale bar = 200 μm. (B) n = 5, three fields per sample were selected. For all statistical plots, values are expressed as mean ± SD. ***P* < 0.01, *****P* < 0.0001. Statistical significance was determined using Student's t-test (for A, B, and C). CSA, cross-sectional area

**Supplementary Figure 3. Keratocan is upregulated during C2C12 myoblast differentiation.** (A) Western blotting and quantitative analysis of keratocan protein expression levels in GM and DM groups, n = 3. (B) Representative IF staining and fluorescence intensity of keratocan in GM and DM groups; scale bar = 200 μm, n = 5, three fields per sample were selected. (C) Representative IF staining of overlapping fluorescence of keratocan and MyHC four days after differentiation. For all statistical plots, values are expressed as mean ± SD. ** *P* < 0.01, **** *P* < 0.0001. Statistical significance was determined using Student's t-test (for A and B). GM, growth medium; DM, differentiation medium

**Supplementary Figure 4. Representative SDH staining in GA muscle of each group and quantification of SDH-positive fibers, scale bar = 100 μm. n = 5, three fields per sample were selected. ** *P* < 0.01, *** *P* < 0.001. Statistical significance was determined using one‐way ANOVA. GA, gastrocnemius. Control, mice injected with the same volume of PBS; AAV9-oe-Scramble, mice injected with scrambled oeRNA vector control; AAV9-oe-Kera, mice injected with AAV9 vectors encoding oe-Kera**

**Supplementary Figure 5. Diagram of the PI3K/AKT signaling pathway**

| **Table S1. Summary of Mouse Sample Characteristics** | | | | | |
| --- | --- | --- | --- | --- | --- |
| **Parameter** | **GSE209528** | **GSE175562** | **GSE213148** | **GSE186104** | **GSE202395** |
| **Tissue** | **TA** | **GA** | **Quad** | **PCF-RMB** | **Bone** |
| **Sex** | **Male** | **Male** | **Male** | **NR** | **Male** |
| **Strain** | **C57BL/6J** | **C56BL/6J** | **C57BL/6J** | **Sprague Dawley** | **Sprague Dawley** |
| **Age** | **Fifteen weeks** | **2 months/12 months** | **4 months/21 months** | **Time-mated rat E19** | **NR** |
| **Treatment** | **NC** **vs DEX** | **Normal diet vs High-fat diet** | **None** | **SCM vs SCM+ DEX** | **Sham operation vs**  **OVX** |
| **Condition** | **NS vs SP** | **NS vs SP** | **NS vs SP** | **NO vs OP** | **NO vs OP** |

**TA, tibialis anterior muscle; GA, gastrocnemius; Quad, quadriceps muscle; PCF-RMB, primary cultured fetal rat metatarsal bones; NR: not reported; NC, normal control; DEX, dexamethason; SCM, standard cultivation medium; OVX, ovariectomized; SP, sarcopenia; NS, non-sarcopenia; OP, osteoporosis; NO, non-osteoporosis; VS, versus**

| **Table S2. Primers used in RT-qPCR experiments** | | |
| --- | --- | --- |
| Name | Forward primer | Reverse primer |
| Keratocan (mus) | 5′-CGTCGAGGGGTTTTGATGTG-3′ | 5′-GCTCAGTTGTGGTCCGTGAA-3′ |
| Syn2(mus) | 5′-CGTGCATGGCAAAGATGGCA-3′ | 5′-TCAGTGATGAGTTGTCTGTCCT-3′ |
| GAPDH (mus) | 5′-TGGAAAGCTGTGGCGTGATG-3′ | 5′-TACTTGGCAGGTTTCTCCAGG-3′ |

| **Table S3. Antibodies and their application** | | | | |
| --- | --- | --- | --- | --- |
| Antibody name | Catalog Number | Source | Dilution ratio | |
|  |  |  | WB | IF |
| Anti-GAPDH antibody | HRP-60004 | Proteintech | 1/10000 |  |
| Anti-MyoD1 antibody | ab203383 | Abcam | 1/100 |  |
| Anti-MyoG antibody | ab124800 | Abcam | 1/200 | 1/500 |
| Anti-Fast Myosin Skeletal Heavy chain antibody | ab91506 | Abcam | 1/1000 | 1/1000 |
| Anti-Ki67 antibody | A16919 | ABclonal | 1/1000 | 1/200 |
| Anti-MuRF-1 antibody | A3101 | ABclonal | 1/2000 |  |
| Anti-Atrogin-1 antibody | A3193 | ABclonal | 1/2000 |  |
| Anti-Myostatin antibody | A22725 | ABclonal | 1/2000 |  |
| Anti-Keratocan antibody | AP12617b | Abcepta | 1/1000 | 1/200 |
| Anti-AKT antibody | 60203-2-Ig | Proteintech | 1/5000 |  |
| Anti- Phospho-AKT1-S473 antibody | AP0637 | ABclonal | 1/1000 |  |
| Anti- PI3K antibody | A22996 | ABclonal | 1/1000 |  |
| Anti-Phospho-PI3KP85α/γ/β-Y467/Y199/Y464 antibody | AP0854 | ABclonal | 1/2000 |  |
| Anti-mTOR antibody | A2445 | ABclonal | 1/1000 |  |
| Anti-Phospho-mTOR-S2448 antibody | AP0115 | ABclonal | 1/1000 |  |
| Anti-Lamin B1 antibody | A11495 | ABclonal | 1/1000 |  |
| Anti-CCND1 antibody | 380999 | Zenbio | 1/1000 | 1/100 |
| Anti-Rabbit IgG(H+L) HRP | GAR007 | MultiSciences | 1/100000 |  |
| Anti-Mouse IgG(H+L) HRP | GAM007 | MultiSciences | 1/100000 |  |
| Anti-PCNA antibody | A9909 | ABclonal | 1/1000 | 1/200 |
| Anti-Pax7 antibody | bs-22741R | Bioss |  | 1/100 |
| Anti-Fast Myosin Skeletal Heavy chain antibody | GB112130 | Servicebio |  | 1/1000 |
| Anti-Slow Myosin Skeletal Heavy chain antibody | GB112131 | Servicebio |  | 1/1000 |
| laminin | Ab11575 | Abcam |  | 1/50 |
| Anti-Rabbit IgG(H+L) (Alexa Fluor 594) | ab150084 | Abcam |  | 1/500 |
| Anti-Rabbit IgG(H+L) (Alexa Fluor 488) | ab150077 | Abcam |  | 1/500 |
| Myosin heavy chain Type I（MYH7） | BA-D5 | DSHB |  | 1/200 |
| Myosin heavy chain Type IIA（MYH2） | SC-71 | DSHB |  | 1/200 |
| Myosin heavy chain Type IIB（MYH4） | BF-F3 | DSHB |  | 1/200 |
| Goat anti-Mouse IgG2b Cross-Adsorbed Secondary Antibody（Alexa Fluor™ 350） | A-21140 | Invitrogen |  | 1/500 |
| Goat anti-Mouse IgG1 Cross-Adsorbed Secondary Antibody（Alexa Fluor™ 488） | A-21121 | Invitrogen |  | 1/500 |
| Goat anti-Mouse IgM Cross-Adsorbed Secondary Antibody（Alexa Fluor™ 555） | A-21426 | Invitrogen |  | 1/500 |

| **Table S4. The sequences of sh-RNAs** | |
| --- | --- |
| Name | Sequence |
| sh-Kera | 5′-GCCAACACCATGCAACTCTTT-3′ |
| Sh-NC | 5′-TTCTCCGAACGTGTCACGT-3′ |

| **Table S5. Clinical characteristics of patients with or without Osteosarcopenia** | | | |
| --- | --- | --- | --- |
| Characteristic | Non-osteosarcopenia  （n = 5） | osteosarcopenia (n = 5） | P‐value |
| Age (years) | 32.2 ± 1.9 | 76.6 ± 2.7 | < 0.0001 |
| Sex | Male: 3, Female: 2 | Male: 2, Female: 3 | > 0.05 |
| Basal Metabolic Rate (kJ/m²·h) | 1314.49 ± 45.34 | 1149.73 ± 84.57 | 0.005 |
| BMI（Kg/m^2^） | 25.3 ± 1.7 | 20.5 ± 1.5 | 0.0013 |
| SMI（Kg/m^2^） | 7.96 ± 1.05 | 5.03 ± 0.81 | 0.0011 |
| Hand grip strength（Kg） | 35.8 ± 2.7 | 14.2 ± 2.0 | < 0.0001 |
| Walking speed（m/s） | 1.4 ± 0.1 | 0.8 ± 0.1 | < 0.0001 |
| FTSST（s） | 8.82 ± 1.18 | 15.56 ± 1.59 | < 0.0001 |
| DXA（g/cm²） | 2.28 ± 0.50 | -3.46 ± 0.42 | < 0.0001 |

BMI: Body mass index; SMI: Skeletal Muscle Mass Index; FTSST: Five-times sit-to-stand test;

DXA: Dual-energy X-ray absorptiometry. The participants were all female.

**Supplemental Methods**

**Micro-computed tomography (µCT)**

Dissected femoral specimens were scanned using a Skyscan 1275 micro-CT (Bruker, USA) at a voltage of 46 kVp, a current of 75 μA, and 10.0 μm pixels. Three-dimensional analysis was performed using CTAn software (Bruker, USA); the parameters analyzed included bone surface area/bone volume (BS/BV), bone surface area/total bone volume (BS/TV), bone volume/total bone volume (BV/TV), number of trabeculae (Tb.N), trabecular thickness (Tb.Th), and bone mineral density (BMD).

**Clinical tissue specimens**

This study was approved by the hospital Ethics Committee (Approval No. K2023-06-008), and all the enrolled patients provided informed consent. Patients who underwent elective hip surgery between June 1, 2023, and December 31, 2023, were selected, regardless of sex, with the following inclusion criteria: 1) age 20–90 years; 2) diagnosed with a femoral neck fracture, ischemic necrosis of the femoral head, congenital hip dysplasia, osteoarthritis, or rheumatoid arthritis with fracture fixation or arthroplasty. The exclusion criteria were as follows: 1) severe hypertension, diabetes mellitus, heart disease, malignancy, or other organ failure; 2) thrombosis or a history of thrombosis within the last 6 months; 3) lower extremity nerve and muscle lesions.

We recorded the age, sex, height, weight, handgrip strength, 6 m walk speed, skeletal muscle mass index, and bone mineral density of all eligible patients. The patients were divided into two groups: an SP OP group and a non-SP OP group. Patients with SP were diagnosed according to the criteria of the Asian Working Group for Sarcopenia. The diagnosis of SP was based on the following: muscle grip strength (male: < 28 kg; female: < 18 kg), 6 m walk speed (< 1.0 m/s), and skeletal muscle content of the extremities DXA (male: < 7.0 kg/m^2^; female: < 5.4 kg/m^2^). Patients with OP were diagnosed according to the World Health Organization criteria, which defines OP as bone mineral density measured by DXA 2.5 standard deviations below the peak bone mass of healthy young people of the same sex and race.

**Cell count kit 8 method (CCK-8)**

Cell proliferation was detected by staining with Cell Counting Kit-8 (C0038, Dojindo, Japan). Briefly, 1000 C2C12 cells were seeded into 96-well plates. In total, 10 μL of the CCK-8 solution was added to each well and then incubated at 37 °C for 1 h. The absorbance of each well was measured at 450 nm using an enzyme marker. The CCK-8 assay was performed at 0, 24, 48, 72, and 96 h after inoculation. All experiments were performed in triplicate.

**5-ethynyl-2'-deoxyuridine (EDU) cell proliferation assay**

In total, 10μM of EDU medium was prepared according to the manufacturer's instructions (C10310-3, C10310-1, RiboBio, China). C2C12 cells were seeded in 24-well plates. After the C2C12 cells reached the appropriate fusion level, the culture medium was changed, and 100 μL of EDU was added. Cells were incubated in 5% CO_2_ at 37 °C for 2 h. Cells were then fixed with 4% paraformaldehyde for 20 min, incubated with Apollo® reagent (100 μL) for 30 min at room temperature, then stained with DAPI dye and observed under a fluorescence microscope. The ratio of EdU-positive cells to the total number of DAPI-positive cells was calculated to determine cell proliferation.

**Determination of muscle fiber-type composition**

**The blood on the muscle surface was washed with PBS, dried with absorbent paper, rapidly frozen, and embedded in a small box containing an OCT embedding medium. Cross-sections from the mid portions of the muscle were cut at 10 µm in a cryostat (Leica Microsystems, Wetzlar, Germany) maintained at -20 °C. The tissues were then permeabilized for 10 minutes in PBS containing 0.3% Triton, followed by sealing in 10% goat serum for 1 hour. They were simultaneously incubated with BA-D5 (type I, mouse antibody IgG2b, myh7, 1:200), SC-71 (type IIa, mouse antibody IgG1, myh2, 1:200) and BF-F3 (type IIb, mouse antibody IgM, myh4, 1:200) overnight at 4 ℃. On the second day, three corresponding fluorescent secondary antibodies were added and incubated for 2 hours at room temperature. We used a variety of secondary antibodies, such as goat anti-mouse IgG2b cross-adsorbed secondary antibody, Alexa Fluor™ 350 (A-21140, Invitrogen, 1:500, blue, type I), goat anti-mouse IgG1 cross-adsorbed secondary antibody, Alexa Fluor™ 488 (A-21121, Invitrogen, 1: 500, green, type IIa), goat anti-mouse IgM (heavy chain) cross-adsorbed secondary antibody, Alexa Fluor™ 555 (A-21426, Invitrogen, 1:500, red, type IIb). Samples were observed by fluorescence microscopy and analyzed using ImageJ software.**

**According to the fluorescence intensity of each MyHC subtype, muscle fiber phenotypes were classified as type I, type IIa, and type IIb, and those not labeled by these antibodies were classified as type IIx fibers. For fiber type analysis, all fibers within the entire muscle cross-section were characterized. We determined the relative proportions of different muscle fiber types by calculating the proportion of positive fibers in slow muscle (type I) and fast muscle (type II).**

**Supplementary references**

S1. Pette D, Staron RS. Myosin isoforms, muscle fiber types, and transitions. Microsc Res Tech 2000; 50(6):500-9.

S2. Neunhäuserer D, Zebedin M, Obermoser M, Moser G, Tauber M, Niebauer J, et al.Human skeletal muscle: transition between fast and slow fibre types. Pflugers Arch 2011;461(5):537-43.

S3. Xiao L, Liu J, Sun Z, Yin Y, Mao Y, Xu D, et al. AMPK-dependent and -independent coordination of mitochondrial function and muscle fiber type by FNIP1. PLoS Genet 2021;17(3):e1009488.

S4. Bahn YJ, Yadav H, Piaggi P, Abel BS, Gavrilova O, Springer DA, et al. CDK4-E2F3 signals enhance oxidative skeletal muscle fiber numbers and function to affect myogenesis and metabolism. J Clin Invest 2023;133(13):e162479.

S5. Wang T, Xu YQ, Yuan YX, Xu PW, Zhang C, Li F, et al. Succinate induces skeletal muscle fiber remodeling via SUNCR1 signaling. EMBO Rep 2019;20(9):e47892.

S6. Pereyra AS, Lin CT, Sanchez DM, Laskin J, Spangenburg EE, Neufer PD, et al. Skeletal muscle undergoes fiber type metabolic switch without myosin heavy chain switch in response to defective fatty acid oxidation. Mol Metab 2022;59:101456.
